# Transplantation of Endothelial Progenitor Cells in Obese Diabetic Rats Following Myocardial Infarction: Role of Thymosin Beta-4

**DOI:** 10.3390/cells9040949

**Published:** 2020-04-12

**Authors:** Kian Keong Poh, Poay Sian Sabrina Lee, Andie Hartanto Djohan, Mary Joyce Galupo, Geronica Gorospe Songco, Tiong Cheng Yeo, Huay Cheem Tan, Arthur Mark Richards, Lei Ye

**Affiliations:** 1 Department of Cardiology, National University Heart Centre Singapore, National University Health System, Singapore 119074, Singapore; kian_keong_poh@nuhs.edu.sg (K.K.P.); ashsabby@gmail.com (P.S.S.L.); andie_hartanto_djohan@nuhs.edu.sg (A.H.D.); mary_joyce_galupo@nuhs.edu.sg (M.J.G.); geronica_gorospe_songco@nuhs.edu.sg (G.G.S.); tiong_cheng_yeo@nuhs.edu.sg (T.C.Y.); huay_cheem_tan@nuhs.edu.sg (H.C.T.); mdcarthu@nus.edu.sg (A.M.R.); 2 Department of Medicine, Yong Loo Lin School of Medicine, National University of Singapore, Singapore 117597, Singapore; 3 National Heart Research Institute Singapore, National Heart Centre Singapore, Singapore 169609, Singapore

**Keywords:** endothelial progenitor cells, thymosin beta, myocardial infarction

## Abstract

Endothelial progenitor cells (EPCs) are bone-marrow derived cells that are critical in the maintenance of endothelial wall integrity and protection of ischemic myocardium through the formation of new blood vessels (vasculogenesis) or proliferation of pre-existing vasculature (angiogenesis). Diabetes mellitus (DM) and the metabolic syndrome are commonly associated with ischemic heart disease through its pathological effects on the endothelium and consequent endothelial dysfunction. Thymosin-β4 (Tβ4) which expressed in the embryonic heart is critical in epicardial and coronary artery formation. In this study, we explored the effects of Tβ4 treatment on diabetic EPCs in vitro and intramyocardial injection of Tβ4-treated and non-Tβ4 treated EPCs following acute myocardial infarction (MI) of diabetic rats in vivo. It was found that 10 ng/mL Tβ4 increased migration, tubule formation, and angiogenic factor secretion of diabetic EPCs in vitro. In vivo, although implantation of Tβ4 treated diabetic EPCs significantly increased capillary density and attracted more c-Kit positive progenitor cells into the infarcted hearts as compared with implantation of non-Tβ4 treated diabetic EPCs, the significantly improved left ventricular ejection fraction was only found in the rats which received non-Tβ4 treated EPCs. The data suggests that a low dose Tβ4 increases diabetic EPC migration, tubule formation, and angiogenic factor secretion. However, it did not improve the effects of EPCs on left ventricular pump function in diabetic rats with MI.

## 1. Introduction

Diabetes mellitus is a common metabolic disease with a high and growing prevalence affecting 4% of the population [1]. Around 171 million people were affected in 2000 and 366 million are expected in 2030 worldwide [1]. Diabetes mellitus (DM) causes endothelial dysfunction and is associated with an increased risk of cardiovascular disease [2]. Endothelial dysfunction comprises several malfunctions in the vascular endothelium and plays a primary role in the development of vascular complications associated with diabetes and cardiovascular disease [3]. Hyperglycemia, dyslipidemia, hyperinsulinemia, and inflammation are characteristic features of both types 1 and 2 diabetes. These factors play pivotal roles in diabetes-associated vascular complications, which are the principal causes of morbidity and mortality in patients with diabetes mellitus and ischemic heart disease [4].

Studies demonstrated that high glucose levels (33 mM) increases apoptosis of endothelial progenitor cells (EPCs) in vitro through inhibiting the PI3-kinase/Akt pathway [5]. Inhibited Akt activity decreases the proliferation of EPC. Hypercholesterolemia is demonstrated to be associated with reduced EPC numbers in patients [5], and inhibits EPC proliferative capacity, migratory activity, and in vitro angiogenesis. One possible underlying mechanism is an increased rate of EPC senescence/apoptosis, as oxidized low-density lipoprotein induces endothelial progenitor cell senescence [6].

A pleiotropic factor targeting a multitude of the pathophysiological basis of cardiac ischemia is Thymosin-β4 (Tβ4), which is a 5 kDa polypeptide composed of ubiquitous 43 amino acids [7] and can be found universally in tissues and circulating cells, except red blood cells [8]. It is the most abundant member of the β-thymosin family in mammalian tissue and is regarded as the main G-actin sequestering peptide [7]. Tβ4 plays an important role in promoting cell survival, migration, and proliferation, and specifically promotes cardiac cell survival, migration, and proliferation after an episode of myocardial infarction, thus assisting in improving post-infarction myocardial function [9,10]. It has been suggested that these anti-apoptotic properties displayed by Tβ4 is mediated by activating integrin-linked kinase (ILK), which results in activation of the survival kinase, Akt [8,9].

Tβ4 also possesses angiogenic activity which is believed to be attributed to its seven amino acid actin binding motifs. It has been demonstrated to promote endothelial cell migration and adhesion, tubule formation, aortic ring sprouting, and angiogenesis [11]. Indeed, it has also been reported to confer cardioprotection mediated by EPCs [10]. Tβ4 also promotes epicardial progenitor cells differentiation into endothelial cells, thereby serving as a source of vascular progenitors for coronary vasculogenesis and angiogenesis [12]. Though the effects of Tβ4 have been widely studied, its effect on EPCs derived from diabetic subjects is not yet known. In this study, we investigated the effects of Tβ4 on EPCs derived from diabetic rats.

## 2. Materials and Methods

### 2.1. Animals

Animal experiments were approved by the Institutional Animal Care and Use Committee, National University of Singapore and followed the Guide for the Care and Use of Laboratory Animals. Zucker diabetic fatty rats (*n* = 28, ZDF/Gmi-*fa/fa,* Charles River Laboratories, Wilmington, MA, USA) of 20 weeks of age were used for EPC isolation in vitro (*n* = 8) and in vivo transplantation studies (*n* = 20). All animals were housed individually in automated ventilated cages in environmentally controlled rooms (22–24 °C; 50–70% relative humidity) with a 12-h light/dark cycle. All animals were maintained on Purina 5008 diet (LabDiet, St. Louis, MO, USA) as recommended by the supplier. The composition of this diet by weight is 23% protein, 58.5% carbohydrate, and 6.5% fat.

### 2.2. Peripheral Blood Mononuclear Cell Isolation and Culture

Rats (*n* = 8) were anaesthetized by isoflurane (4%) inhalation. The rats were then euthanized via intraperitoneal injection of pentobarbital (30 mg/kg). Blood (approximately 8 to 10 mL per rat) was collected from cardio-puncture into an EDTA vacutainer tube. Blood was processed to isolate mononuclear cells within two hours after collection. 

Mononuclear cells were isolated from rat peripheral blood by density gradient centrifugation using Ficoll-Paque™ Premium 1.084 (GE Healthcare, Little Chalfont, UK). Briefly, Ficoll-Paque™ (10 mL) was added to a 50 mL Falcon tube. Blood (8–10 mL) was diluted with phosphate buffered saline (PBS) without Ca/Mg in ratio of 1:1 and then carefully layered on top of the Ficoll-Paque™. Cells were centrifuged at 300× *g* for 35 min at room temperature without the brake. The upper plasma layer was removed without disturbing the plasma-Ficoll-Paque™ and washed twice with PBS without Ca/Mg. Generally, 3 × 10^6^ mononuclear cells would be isolated from 10 mL blood and cultured on wells of BioCoat™ fibronectin-coated 24-well plate in endothelial growth media-2 (EGM-2) (Lonza, Switzerland) bullet kit which consists of endothelial basal medium (EBM, Lonza, Switzerland) supplemented with 0.5mL single aliquots of recombinant human epidermal growth factor (rhEGF), vascular endothelial growth factor (VEGF), ascorbic acid, heparin, gentamicin + amphotericin-B (GA-1000), hydrocortisone, human fibroblast growth factor-B (hFGF-B), insulin growth factor-1 (IGF-1), and 20% fetal bovine serum (FBS) (Lonza, Switzerland) for 7 days. Non-adherent cells would be removed after day 4. Attached cells would be harvested to isolate EPCs by fluorescence-activated cell sorting (FACS).

### 2.3. EPC Isolation and Culture

Attached mononuclear cells on day 7 were harvested for EPC isolation. This was performed by cell sorting based on CD34^+^ and KDR^+^ cell surface markers. Briefly, 3 × 10^6^ attached mononuclear cells were incubated initially with FcR blocking reagent (Miltenyi Biotec, Germany) and then stained with anti-mouse/rat CD34^+^ conjugated with Phycoerythrin (PE) (R&D Systems, Minneapolis, MN, USA). This was followed by anti-VEGF receptor 2 (VEGFR2) antibody (KDR/EIC) conjugated with Fluorescein isothiocyanate (FITC) (Abcam, Cambridge, UK). The respective isotype antibodies served as controls. After incubation, cells were washed with PBS containing 2% FBS, re-suspended in 0.3 mL 2% FBS/PBS containing 5 µL of propidium iodide (10 μg/mL). The cell sorting is under Single Cell Mode using BD FACS Calibur Flow Cytometer 2 Laser 4 Color with FACS Loader (BD Biosciences). A low flow rate of 300–400 events/sec was used. Isotype control sample was used as negative control. Live cells of adequate size and granularity which were positive for both VEGFR2 (FITC) and CD34^+^ (PE) were collected [13]. Data were processed using the BD CellQuest Pro software (version 5.1). Generally, we obtained 1 × 10^6^ CD34^+^KDR^+^ EPCs from 3 × 10^6^ attached mononuclear cells. Isolated EPCs were cultured in EGM-2 medium for 7 days before being used for subsequent experiments.

### 2.4. Migration and Tubule Formation Assays

Isolated EPCs were cultured for 7 days and detached using 1 mmol/L EDTA in PBS (pH 7.4), harvested by centrifugation, and resuspended in 500 µL EBM. 1 × 10^4^ EPCs were placed in the upper chamber of a sterile 6.5mm Transwell® with 3.0 µm pore. The chamber was placed in a 24-well plate containing EBM supplemented with 50 ng/mL mouse vascular endothelial growth factor (VEGF) (Sigma-Aldrich, USA) and with or without 10 ng/mL Tβ4. After 24-h incubation at 37 °C, the lower side of the filter was washed with PBS and fixed with 2% formaldehyde. Cells migrating into the lower chamber were counted manually in 4 random microscopic fields. For tubule formation assay, 100 µL ECM gel from Engelbreth Holm-Swarm mouse sarcoma (Sigma-Aldrich) was layered into 96 well plates for 1 h at 37 °C. 1 *×* 10^4^ EPCs were seeded into each well and cultured with EBM supplemented with 50 ng/mL mouse VEGF with or without 10ng/mL Tβ4 at 37 °C for 8 h. Incorporated cells were counted from 4 random microscopic fields per rat. In brief, prior to the quantification, 1mm by 1mm grid area has been drawn onto each well. Tubule formation are measured by the total tube length of tubes in each microscopic field. The average length of the tubes was calculated from 4 microscopic fields and finally divided by the grid area of the wells covered by the microscopic fields. All the measurements of the tubule formation were quantified using Image J software (version 1.50a).

### 2.5. Quantification of Paracrine Factors Released by EPCs

1 × 10^5^ EPCs were cultured for 72 h under hypoxic conditions (1.5% O_2_, 5% CO_2_, 93% N_2_) in a humidified gas-sorted hypoxic incubator using EBM with 1% FBS, treated with and without 10 ng/mL Tβ4 for 5 days. EPC conditioned medium was centrifuged and sterile filtered with a 0.22 µm and stored at −80°C until use. The concentration of platelet-derived growth factor-BB (PDGF-BB) (Rat PDGF-BB Quantikine ELISA kit, R&D Systems), IGF-1 (Rat IGF-1 Quantikine ELISA kit, #MG100; R&D Systems), and VEGF (Rat VEGF Quantikine ELISA kit, #RRV00; R&D Systems) in treated and non-treated EPCs was assessed by the Bioplex system (Bio-Rad) following the manufacturer’s instructions. Cells were also harvested for gene expression using real time-PCR, and total RNA was isolated using Trizol. cDNA was made from 5 µg total mRNA using a 1st strand cDNA synthesis kit (Invitrogen) at 25 °C for 5 min, at 50 °C for 60 min, and a 70 °C for 15 min. Then, PCR amplification was performed for VEGF, IGF-1, and PDGF-BB with TaqDNA polymerase. The following primer sets were used: VEGF, forward 5′-GACAAGATGGTGAAGGTCGGT-3′, and reverse 5′-AGGGTAAGCCACTCACACACA-3′; PDGF-BB, forward 5′-TGAAATGCTGAGCGACCAC-3′and reverse 5′-AGCTTTCCAACTCGACTCC-3′; IGF-1, forward 5′-GCATTGTGGATGAGTGTTGC-3′ and reverse 5′-GGTCTTGTTTCCTGCACTTC-3′. PCR was performed with the following cycles: 40 cycles of 10 s at 95 °C, 30 s at 62 °C, and 30 s at 70 °C. Reactions were performed using SYBR-Green PCR Master Mix.

### 2.6. Rat Heart Model of Myocardial Infarction and EPC Transplantation

To determine the biological function in vivo, 10 ng/mL Tβ4-treated and non-treated EPCs will be directly injected into hearts of ZDF rats (*n* = 20) after myocardial infarction (MI) [14,15]. The animals were anesthetized with Ketamine/Xylazine mixture, intubated, and mechanically ventilated with a rodent ventilator. Considering that Isoflurane can decrease both systolic and diastolic function with a greater reduction in cardiac output, peak aortic flow, and higher left ventricular end-diastolic pressures [16], we did not use isoflurane for induction of anesthesia and echocardiography. Instead, we used ketamine/xylazine mixture for both induction of and maintenance of anesthesia.

A left-side thoracotomy was performed to expose the heart and the pericardial layer was removed. Experimental acute MI was induced by permanent ligation of the left anterior descending artery. This was followed by randomization of ZDF rats to experimental groups ten minutes following induction of MI: Tβ4-treated EPCs (*n* = 7), non-Tβ4 treated EPCs (*n* = 6), and medium alone injected (*n* = 6). 0.2 mL Dulbecco’s Modified Eagle’s Medium (DMEM) alone or containing 1 × 10^6^ Tβ4-treated or non-Tβ4 treated EPCs were injected intra-myocardially into left ventricular (LV) anterior wall. After surgery, the rats were treated with Baytril to prevent infection and ketoprofen for analgesia. The rats will then be euthanized, and hearts explanted for immunohistochemistry studies 6 weeks post-surgery.

### 2.7. Echocardiographic Protocol

Echocardiography was performed 6 weeks after induction of MI. All the rats were anesthetized with ketamine/xylazine mixture and positioned in left lateral decubitus where the anterior chest hair was shaved, electrocardiogram (ECG) was monitored throughout the experiment and temperature was maintained at 37 °C on the heated plate. The examination was performed using the Vivid 7 Dimension ultrasound system/broadband 10S with a frequency range of 4.0 to 10.5 MHz (GE Healthcare). Left ventricular (LV) structure and function were assessed from short axis (SAX) views (at the level of the papillary muscles), and from the 4-chamber apical windows. Mitral inflow was recorded by pulsed wave Doppler. Measurements were obtained from three consecutive cardiac cycles by off-line analysis (EchoPAC, GE Healthcare). Left-ventricular end-systolic and end-diastolic internal diameters (LVIDes and LVIDed) were determined from M-mode images in the SAX view. LV ejection fraction (LVEF) was calculated according to the following formulas: LVEF = 1 − (LVIDes/LVIDed)^2^ [15]. LV systolic (S’) and early diastolic (E’) myocardial velocity was obtained from on-line tissue Doppler (TD) recordings at the level of the septal mitral annulus. Images were acquired at a frame rate of 116 frames/s, with optimal sector depth and width. Global circumferential and radial systolic strain (GCS, GSR) and strain rate (GSrC, GSrR), rotation (Rot) and rotation rate (RotR) were calculated. All measurements were performed by the same single observer.

### 2.8. Immunohistochemistry Assessments

Following euthanasia, rat hearts were explanted and cut into 5-µm paraffin-embedded sections. Vasculogenesis was evaluated by staining sections with rabbit anti-CD31^+^ primary antibodies (1:100; Santa Cruz Biotech), FITC-conjugated donkey anti-rabbit IgG secondary antibodies (JacksonImmuno Research, West Grove, PA, USA) and tetramethylrhodamine (TRITC)-conjugated anti-smooth-muscle actin antibodies (SMA, 1:500; Sigma-Aldrich). Vascular structures positive for CD31^+^ expression (i.e., FITC fluorescence), and for both CD31^+^ and SMA expression (i.e., simultaneous FITC and TRITC fluorescence) were counted for 6–7 animals per group, in 5–6 slides per animal and 8–10 fields per slide.

The recruitment of endogenous c-Kit^+^ cardiac progenitor cells (CPCs) was evaluated by staining sections with goat anti-c-Kit^+^ antibody (R&D systems), cardiac cells were identified by staining sections with rabbit antibodies against cardiac troponin I (cTnI, Abcam). c-Kit^+^ cells were counted for 6–7 animals per experimental group, in 5–6 slides per animal and 4–5 fields per slide.

### 2.9. Statistical Analysis

Data are expressed as mean ± SD. Comparisons of number and function of EPCs between Tβ4-treated and non-Tβ4 treated EPCs were performed using unpaired t-tests. The effect of Tβ4 in the rat study was analyzed using 1-way ANOVA with LSD correction. All statistical data was performed using SPSS. Statistical significance is assumed if *p* value < 0.05.

## 3. Results

### 3.1. Tβ4 Treatment Increased Diabetic EPC Migration and Tubule Formation

**Figure 1:**
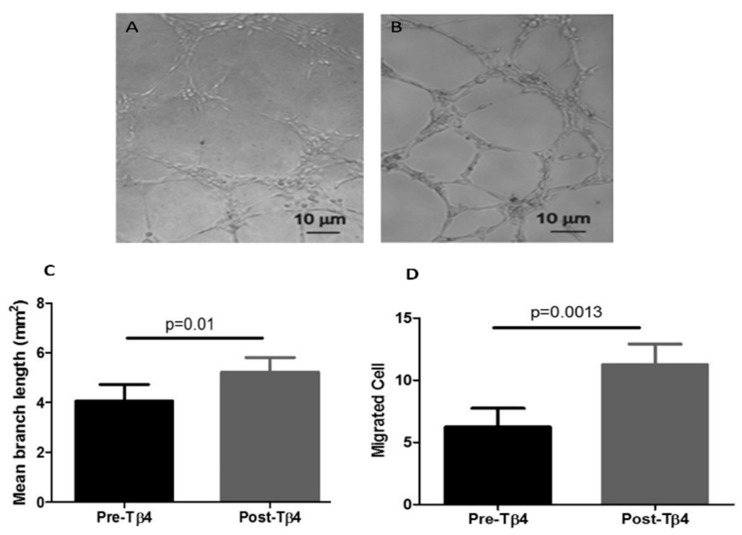
Function of EPCs in vitro. Representative images of tubule formation on Matrigel by non-Tβ4 treated (**A**) and Tβ4-treated (**B**) EPCs. (**C**) Quantification of mean branch length of tubules. (**D**) Quantification of EPCs in migration assay (Magnification of A&B = 40×) (*n* = 8).

It was found that 10 ng/mL Tβ4 significantly enhanced tubular formation capability of EPCs derived from ZDF rats (Figure 1A–C). The branch length formed by Tβ4-treated EPCs was increased by 25% as compared with non-Tβ4 treated EPCs. Furthermore, 10 ng/mL Tβ4 treatment significantly enhanced migration capability of diabetic EPC by 50% as compared with non-Tβ4 treated EPCs (Figure 1A–D).

### 3.2. Tβ4 Treatment Increased the Expression of Angiogenic Growth Factors from EPCs Isolated from ZDF Rats

**Figure 2:**
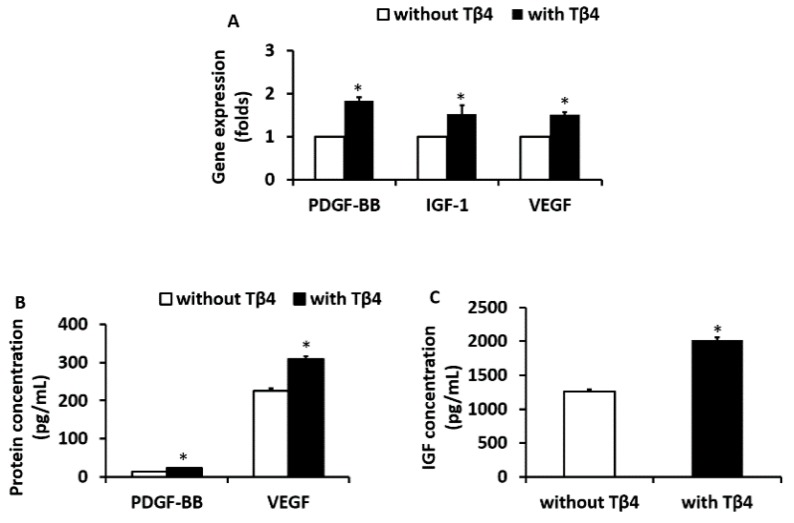
(**A**) Gene expression levels of platelet derived growth factor-BB (PDGF-BB), insulin like growth factor-1 (IGF-1), and vascular endothelial growth factor (VEGF) from Tβ4-treated and non- Tβ4 treated EPCs. Protein concentrations of PDGF-BB and VEGF (**B**) or IGF-1 (**C**) from Tβ4-treated and non-Tβ4 treated EPCs as determined by respective ELISA kit (*n* = 8) (*: *p* < 0.05, vs. non-Tβ4 treated EPCs).

Tβ4 (10 ng/mL for 5 days) treated diabetic EPCs had significantly increased gene and protein expression levels of PDGF-BB, IGF-1, and VEGF compared to non-Tβ4 treated EPCs (Figure 2A–C), suggesting that Tβ4 is able to enhance the angiogenic function of EPCs derived from ZDF rats.

### 3.3. EPC but not EPC + Tβ4 Treatment Improved Cardiac Function

**Figure 3:**
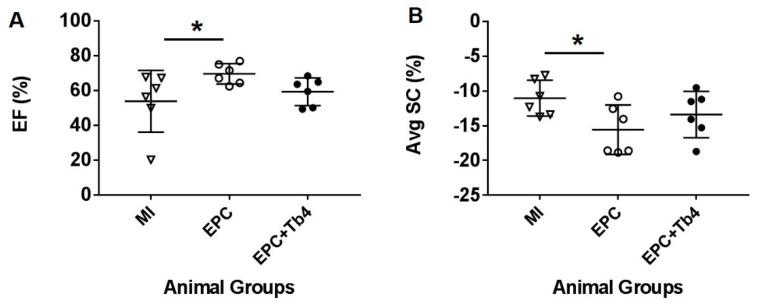
Dot-plot of ejection fraction (EF) (**A**) and average myocardial circumferential systolic strain (AvgSc) (**B**) of each rat in the MI, EPC, and EPC + Tb4 Groups. (One-way ANOVA, * *p* < 0.05).

There was no significant difference in left ventricular (LV) dimensions between the control rats, EPC-transplanted, and EPC + Tβ4-transplanted rats with post-MI and treatment. However, LVEF was significantly improved in the EPC Group, but not in the EPC + Tβ4 Group, as compared to the control Group (Figure 3 and Table 1). The circumferential strain was improved only in the EPC Group as compared to the control Group (−15.6 ± 3.6% vs. −11.0 ± 2.6%; Table 1). In addition, mitral annular tissue Doppler, speckle tracking radial and rotational parameters were not significantly different between groups (Table 1).

### 3.4. Tβ4 Treatment EPCs Increased Angiogenesis Post-Myocardial Infarction (MI)

**Figure 4:**
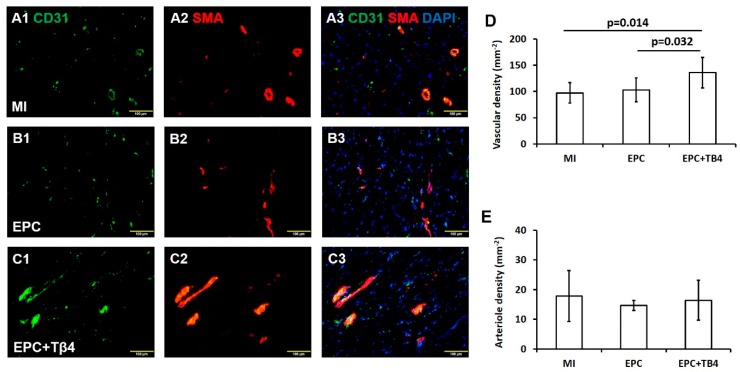
Fluorescence immunostaining for CD31^+^ (**A1**, **B1**, and **C1)** and smooth muscle actin (SMA) (**A2, B2,** and **C2**) expressions in hearts of MI, EPC, and EPC + Tβ4 rat groups. (**A3**) Overlay images of A1 and A2. (**B3**) Overlay images of B1 and B2. (**C3**) Overlay images of C1 and C2. Quantification of total vascular density based on CD31^+^ expression (**D**) and arteriole density based on co-localization of CD31^+^ and SMA (**E**) (A–C magnification = 200×) (*n* = 6 for each group).

Vascular density and arteriole density at border zone of the infarct were calculated based on endothelial-cell marker CD31^+^ and alpha smooth-muscle actin (SMA) expression (Figure 4A–C). CD31^+^ vessel density was significantly greater in sections from the hearts of EPC + Tβ4 Group rats compared to EPC monotherapy or control MI group (*p* < 0.05) (Figure 4D). Arteriole density (the number of CD31^+^/SMA^+^ vessels) was similar in MI, EPC, and EPC + Tβ4 sections (Figure 4E). Thus, Tβ4 appears to enhance the angiogenic response to EPC transplantation by increasing capillary but not arteriole proliferation.

### 3.5. Tβ4 Treated EPCs Increases the Recruitment of Endogenous Cardiac Progenitor Cells (CPCs) Post-MI

**Figure 5:**
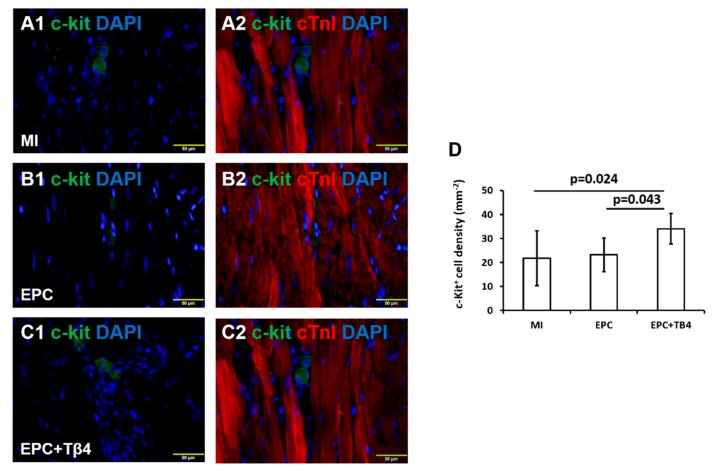
Fluorescence immunostaining for c-Kit (**A1**, **B1**, and **C1**) and cardiac troponin I (**A2**, **B2**, and **C2**) expressions in hearts of MI, EPC, and EPC + Tβ4 rat groups. (**D**) Quantification of c-Kit^+^ cardiac cells in in hearts of MI, EPC, and EPC + Tβ4 rat groups. (A–C magnification = 400×) (*n* = 6 for each group).

Expression of cardiac progenitor cell (CPC) marker c-Kit^+^ was evaluated in the hearts of ZDF rats to determine the effect of intramyocardial injection of Tβ4-treated and untreated EPC. c-Kit^+^ cell density in both infarct and peri-infarct regions was significantly greater in the EPC + Tβ4 Group rats than the EPC Group or the control MI group (Figure 5). No significant differences were observed between the EPC and the control groups, thus suggesting the potential for Tβ4 to recruit endogenous CPC activity.

## 4. Discussion

Endothelial dysfunction plays a primary role in the development of vascular complications associated with diabetes and cardiovascular disease [3,17]. Furthermore, it was also reported that Tβ4 upregulation occurs following myocardial injuries such as MI, providing substrates for neovascularization and paracrine signals for endogenous stem cell recruitment to assist in wound repair [18].

Using EPCs from ZDF rats as cell models, we found that low-dose Tβ4 treatment improved EPC function including migration, tubule formation, and angiogenic cytokine release in vitro. 

It has been shown that Tβ4 plays important roles in promoting cell migration, proliferation and survival [9,11,15,19,20,21,22,23]. It enhances cardiac cell survival, migration, and proliferation [19]. Furthermore, Tβ4 promotes angiogenesis, vasculogenesis [11,21], and enhances regenerative potency of transplanted mesenchymal stem cells (MSCs) after MI [15]. Tβ4 also possesses anti-apoptotic and anti-inflammatory properties [24,25,26] by activating Akt through integrin-linked kinase (ILK) [9,27]. As chronic inflammation is a primary driver of atherosclerosis [28], the pleiotropic cardioprotective effects conferred by Tβ4 (including activation of cardiomyocyte survival pathways, inhibiting endothelial apoptosis, reduction of inflammatory cell recruitment) might have a role in therapeutics for both acute and chronic myocardial ischemia, as it addresses the pathophysiological basis of cardiovascular disease [10].

In addition, Tβ4 also possesses angiogenic activity by enhancing endothelial cell differentiation and angiogenesis [29] through Notch signaling [30]. Our study further demonstrated that Tβ4 also enhanced diabetic EPC migration and tubule formation capability by 50% and 25%, respectively, compared with non-Tβ4 treated EPCs, which is shown to be mediated by PI3K/Akt/ endothelial nitric oxide synthase (eNOS) signal pathway [31]. It is known that EPCs-mediated endothelial cell repair is impaired in coronary heart disease [17]. Thus, Tβ4 can be potentially be used to enhance migration of bone marrow derived EPCs to the ischemic myocardium to aid in endothelial cell repair.

Furthermore, Tβ4-treated EPCs are capable of the synthesis and release of paracrine factors such as PDGF-BB, IGF-1, and VEGF, which contribute to angiogenesis and may be responsible for the improved capillary growth as seen in the EPC + Tβ4 transplanted rat myocardium following an acute ischemic insult. Our study showed that total vessel density was significantly increased in the EPC + Tβ4 group only, while no significant difference in arteriole density was observed between the cells in rat myocardium post EPC + Tβ4 transplantation, which may be derived from various potential sources. Lastly, improved c-Kit+ cell density may also contribute to enhanced vessel density observed in EPC + Tβ4 [32] transplanted rat myocardium.

Interestingly, we found that the measured LVEF was highest in the EPC-transplanted group compared to EPC + Tβ4 and control groups. Speckle-tracking strain analyses, a novel implementation on ZDF rats (to the best of our knowledge) further demonstrated that EPC-transplanted group had better circumferential systolic strain compared to EPC + Tβ4 and control groups. Concomitantly, both EPC and EPC + Tβ4 groups had better early diastolic circumferential strain rate compared with control MI group. Radial and longitudinal systolic and diastolic functions as well as rotational parameters were unaffected by EPC transplantation, regardless of treatment with Tβ4. These indicate that although Tβ4 can enhance migration, tubule formation, and angiogenic factor synthesis and release of EPC in vitro, a transient treatment EPC with low dose Tβ4 may be insufficient to sustain long-term enhanced global EPC function. The most sensitive directional strain parameters, circumferential strain, and strain rate suggest subtle improvement in diastolic and systolic functions by EPC transplantation post-MI. It is known that EPCs are dysfunctional in the diabetic state [33]. Hence, although Tβ4-treated EPCs significantly increased capillary density and recruited more c-Kit progenitor cells to infarcted hearts, the function of recruited progenitor cells is impaired due to the inherent hyperglycaemic and hyperlipidaemic state of the ZDF rats. The cardioprotective effects of Tβ4 in ischaemia-reperfusion injury has been previously described in another preclinical animal study [10]. We postulate that the recruitment of progenitor cells with impaired function may blunt the regenerative capacity of EPCs on the post-infarct myocardium, thus leading to a paradoxical reduction in LVEF in the Tβ4-treated EPC group compared to non-Tβ4-treated group.

## 5. Limitations

This study has several limitations. First, we determined the effect of a low dose of Tβ4 which demonstrated beneficial effects on diabetic EPCs in vitro. However, a higher dosage of Tβ4 may be needed for in vivo, which is worthy to be further investigated. Second, the beneficial effects of Tβ4 on diabetic EPCs is inherently limited due to the small sample size (*n* = 6 in each of the 3 groups, which is a minimal number required in rodent studies). Hence it is worthwhile to consider a larger study with higher doses of Tβ4 to study the potential effects of Tβ4 + EPC in the ischemic myocardium of diabetic rats. Third, the use of CD31^+^, c-Kit^+^, and SMA may be insufficient to evaluate the effects of treatment at the tissue level, more proteins commonly expressed by endothelial and smooth muscle cells should have been included in the immunohistochemical analysis. The sole use of c-Kit^+^ as a biomarker of CPC may have led to an overestimated CPC as c-Kit is also expressed by haematopoetic cells. Fourth, the evaluation done in this study were carried out at the peri-infarct area. Future work should evaluate the different areas of the myocardium (infarct, peri-infarct, and healthy) to determine the different effects of intra-myocardial administration of EPCs on non-viable, stunned, or hibernating, and healthy myocardial cells. Fifth, non-diabetic rats should be included as controls to evaluate the potential cardioprotective effects of EPC and Tβ4 administration post-MI in the absence of diabetes as compared to the diabetic state. Lastly, our study shows that low dose Tβ4 decreased myocardial recovery, therefore a larger dose may decrease EPCs’ effect on myocardial recovery even further.

## 6. Conclusions

Though the in vitro results of Tβ4-treated EPCs are promising, these do not translate to an in vivo improvement in cardiac function post-MI. Our results suggest that intramyocardial injection of Tβ4-treated EPCs blunts the therapeutic capacity of EPCs on post-MI recovery due to recruitment of dysfunctional EPCs. Hence, the selection of subjects is of paramount importance in future studies involving use of Tβ4-treated EPCs.

## Figures and Tables

**Table 1:** Effects of transplanted EPC with and without TB4 on echocardiographic parameters.

	MI (*n* = 6)	EPC (*n* = 6)	EPC + TB4 (*n* = 6)
Body weight (g)	403 ± 34.5	423.83 ± 15.2	418.3 ± 4
LVIDd (mm)	8.7 ± 0.83	8.95 ± 1.4	7.9 6 ± 0.4
LVIDs (mm)	5.87 ± 1.43	4.95 ± 1.13	5.04 ± 0.3
LVEF (%)	53.9 ± 17.8	69.6 ± 5.9^#^	59.34 ± 8
Mitral E (cm/s)	109.3 ± 14	105.7 ± 7.3	115.83 ± 58
HR (bpm)	206.3 ± 31.5	226.7 ± 21.1	234.67 ± 40.3
Septal E’ (mm)	62.7 ± 25.7	49 ± 10.5	64.83 ± 45
Septal S’ (mm)	45.2 ± 21.5	45.3 ± 11.6	40 ± 16.89
LAT E’ (mm)	49.5 ± 10.5	43.2 ± 11.1	50.67 ± 35.3
LAT S’ (mm)	36.7 ± 11.1	38 ± 10.5	33.67 ± 17.4
Avg E’ (mm)	56.1 ± 19.9	46.1 ± 10.7	57.8 ± 39.3
Avg S’ (mm)	40.9 ± 16.9	41.7 ± 11.2	36.8 ± 16.7
Avg SC (%)	−11 ± 2.6	−15.6 ± 3.6^#^	−13. 4 ± 3. 3
Avg SR (%)	17.1 ± 8.6	27.7 ± 16.1	22.6 ± 10.4
Avg SrC S (1/s)	−3.49 ± 0.87	−3.64 ± 0.35	−3.64 ± 0.3
Avg SrC E (1/s)	3.82 ± 1.69	4.71 ± 0.82	4.95 ± 0. 0.86
Avg SrC A (1/s)	3.82 ± 1.64	3.59 ± 0.62	3.37 ± 0.7
Avg SrR S (1/s)	4.81 ± 1.26	4.5 ± 0.92	5.14 ± 1.08
Avg SrR E (1/s)	−4.08 ± 3.75	−6.67 ± 2.66	−7.21 ± 1. 36
Avg SrR A (1/s)	−5.26 ± 1.58	−4.19 ± 2.27	−4.18 ± 0.69
Avg Rot S (°)	−2.7 ± 2	−0.09 ± 2.65	0.1 ± 1.5
Avg RotR S (°/s)	−101.8 ± 52.6	−96.7 ± 78.7	−68 ± 18.43
Avg RotR E (°/s)	111.6 ± 52.3	112.6 ± 47.5	118.76 ± 23.08
Avg RotR A (°/s)	119.1 ± 50.2	112.8 ± 42.6	90.22 ± 28.74

LVIDd: the end-diastolic internal dimension; LVIDs: the end-systolic internal dimension; LVEF: Left ventricular ejection fraction; Mitral E: Early diastolic transmitral flow; HR: heart rate; Septal E’: LV early diastolic myocardial velocity at septal mitral annulus; Septal S’:LV systolic myocardial velocity at septal mitral annulus; LAT E’: LV early diastolic myocardial velocity at lateral mitral annulus; LAT S’: LV systolic myocardial velocity at lateral mitral annulus; Avg E’: (Septal E’ + LAT E’)/2; AvgS’: (Septal S’ + LAT S’)/2; Avg SC: Average myocardial circumferential systolic strain; Avg SR: Average myocardial radial systolic strain; Avg SrC S: Average myocardial circumferential strain rate in systole; Avg SrC E: Average myocardial circumferential strain rate in early diastole; Avg SrC A: Average myocardial circumferential strain rate in late diastole; Avg SrR S: Average myocardial radial strain rate in systole; Avg SrR E: Average myocardial radial strain rate in early diastole; Avg SrR A: Average myocardial radial strain rate in late diastole; Avg Rot S: Average myocardial rotation in systole; Avg RotR S: Average myocardial rotation rate in systole; Avg RotR E: Average myocardial rotation rate in early diastole; Avg RotR A: Average myocardial rotation rate in late diastole. ^#^ *p* < 0.05 EPC group vs. myocardial infarction (MI) group.

## Abbreviations

CPCs	Cardiac progenitor cells
DM	Diabetes Mellitus
DMEM	Dulbecco’s Modified Eagle’s Medium
eNOS	Endothelial nitric oxide synthase
FBS	Fetal bovine serum
FITC	Fluorescein isothiocyanate
hFGF-B	Human fibroblast growth factor-B
IGF-1	Insulin growth factor-1
IHD	Ischemic heart disease
mRNA	Messenger ribonucleic acid
MI	myocardial infarction
MSCs	Mesenchymal stem cells
PBS	Phosphate buffered saline
PCR	Polymerase chain reaction
PDGF-BB	Platelet-derived growth factor-BB
rhEGF	Recombinant human epidermal growth factor
RNA	Ribonucleic acid
SMA	Smooth muscle actin
TRITC	Tetramethylrhodamine
VEGF	Vascular endothelial growth factor
